# The Economic Impact of Premarital Screening (PMS) of Sickle Cell Anemia on the Saudi Health System: A Cost Analysis Study

**DOI:** 10.3390/healthcare13172243

**Published:** 2025-09-08

**Authors:** Amal F. Alotaibi, Rami A. Almalki, Mona Y. Alsheikh, Ghufran O. Omran, Hana A. Althobaiti, Wejdan S. AlQurashi

**Affiliations:** 1 Pharmaceutical Practices Department, College of Pharmacy, Umm Al-Qura University, Makkah 21955, Saudi Arabia; hathobaiti@uqu.edu.sa; 2 Pharmaceutical Care Department, King Faisal Hospital, Makkah Healthcare Cluster, Makkah 24382, Saudi Arabia; raaalmalki@moh.gov.sa (R.A.A.); gomran@moh.gov.sa (G.O.O.); 3 Pharmacy Practice Department, Faculty of Pharmacy, King Abdulaziz University, Jeddah 21589, Saudi Arabia; myalsheikh@kau.edu.sa; 4 Internal Medicine Department, Hematology Unit, King Faisal Hospital, Makkah Healthcare Cluster, Makkah 24382, Saudi Arabia; wsalqurashi@moh.gov.sa

**Keywords:** premarital screening, sickle cell disease, economic evaluation, cost analysis

## Abstract

**Background:** In Saudi Arabia, the government has implemented compulsory premarital screening and consultations for high-risk and positive sickle cell results (traits = AS gene and disease = SS gene). However, despite these measures being in place since 2004, there are still cases of children being born with sickle cell disease. This study aims to evaluate the costs associated with the government’s mandatory premarital screening for sickle cell anemia, compare these expenses with those incurred due to high-risk marriages and analyze the government’s healthcare spending on sickle cell anemia management. **Method:** A decision tree model was conceptualized for the purpose of this study to identify the possible paths from the premarital screening (PMS) procedure. A total of 300,000 cases were processed through this decision tree model. **Results:** The annual management costs for children with the probability of having sickle cell disease are estimated to be USD 10,746,450 in the screening arm and USD 40,488,000 in the no-screening arm. These costs vary depending on the genetic combination of the parents. For individuals with the SS/SS gene combination, the estimated cost is USD 8,137,800 per year. When parents have the SS/AS gene combination, the estimated cost is USD 2,071,950 annually. For those with the sickle cell trait combination (AS/AS), the cost is estimated to be USD 536,700 per year. A direct comparison shows a modeled PMS incremental cost is estimated at USD 29,741,550, which is approximately a 73% reduction in healthcare costs. **Conclusions:** The premarital screening for sickle cell disease is not only cost-saving but also shows the potential for significantly reducing healthcare spending related to sickle cell disease in the future.

## 1. Introduction

The Saudi Ministry of Health defines a healthy marriage as a state of agreement and harmony between the spouses in terms of health, psychological, sexual, social, and legal aspects, aimed at creating a healthy family and producing healthy children [1]. Premarital screening (PMS) is considered a cornerstone in the issue of a healthy marriage.

Since the 1970s, PMS has been a normal practice in many countries, such as Italy, Cyprus, Canada, and the UK, where success rates in preventing issues have reached 80–100% [2]. At a regional level, countries like Bahrain, Jordan, the United Arab Emirates, Iran, and Saudi Arabia have implemented mandatory PMS programs aimed at reducing the complications associated with high-risk marriages. It is essential to recognize that the success of PMS programs is significantly tied to considering social, religious, ethnic, and cultural factors [3]. PMS is designed to assess hemoglobinopathies (such as sickle cell anemia and thalassemia) as well as sexually transmitted diseases (including HIV and viral hepatitis B and C) in couples planning to marry. The fundamental purpose for the screening program is to prevent or reduce the transmission of these diseases to cut healthcare expenditure.

Sickle Cell Disease (SCD) is an autosomal recessive illness characterized by the production of defective hemoglobin S and is associated with a high rate of morbidity and mortality. Research indicates that SCD is a reasonably prevalent genetic condition in the Middle East region; however, information about its incidence in Saudi Arabia is limited and likely underestimated. The frequency of SCD significantly varies greatly across the country, with the Eastern province showing the highest prevalence, followed by the Southern areas [4,5,6,7].

In a 5-year study (2011–2015) for the current national PMS program, the prevalence rate per 1000 population for thalassemia was 13.6, with 12.9 for trait and left for the disease. For SCD, the prevalence rate was 49.6, with 45.8 for trait and left for the disease [8,9]. Memish et al. in 2011 and Alsaeed et al. in 2015 highlighted that consanguineous marriage significantly contributes to the incidence of thalassemia in Saudi Arabia [8,9]. Their research emphasizes that consanguineous marriage is widespread in the country due to cultural norms and traditions, posing a considerable risk for the spread of the disease. The prevalence of relative’s marriage is a prominent pattern in Saudi Arabia, which elevates the likelihood of a greater number of newborns being born with hemoglobinopathies. The implementation of PMS has shown success, indicated by a five-fold increase in the proportion of high-risk couples choosing to cancel their marriage [10]; however, there are barriers to canceling the marriage, including cultural pressures, which is considered the main reason for refusing counseling [11,12]. A study evaluating the screening program, published in 2009 by Alswaidi and O’Brien, reported that despite counseling, 77% of infected spouses decided to proceed with their marriage [3].

Premarital screening is currently conducted across 189 governmental and 97 private facilities nationwide [13]. Samples are collected and sent to main regional laboratories. Risk-free applicants receive certificates enabling their marriage, while high-risk couples are referred to counseling clinics for advice, as the decision to marry is not obligatory. If one of the applicants is found to be infected with viral hepatitis or HIV, the other spouse is informed, and if the couple insists on marriage, the healthy spouse is referred to a preventive medicine clinic for further evaluation [12]. However, Abu-Shaheen et al.’s 2020 [14] epidemiological study concluded that despite the existence of the premarital screening and genetic counseling (PMSGC) program for hemoglobinopathies, reducing the incidence of high-risk couple marriages in Gulf Cooperation Council countries remains a persistent challenge. Their study suggests that the PMSGC program should focus more on high-risk areas to raise awareness regarding hemoglobinopathy disorders and the consequences of consanguinity among high-risk couples [14].

The diseases examined through PMS impose a significant financial burden on the healthcare system. In the United States, the estimated incremental economic burden of SCD is approximately USD 2.98 billion per year (inflation-adjusted dollars in 2015). This cost breakdown includes 57% attributed to inpatient costs (average: USD 15,040 per patient), 38% to outpatient costs (average: USD 10,079 per patient), and 5% to out-of-pocket patient expenses (average: USD 1293 per patient) [15]. According to a systematic review study, the annual healthcare expenditures for patients with SCD range from USD 21,819 to USD 80,843 [16]. Another two studies came to a similar conclusion, estimating annual total medical costs about USD 34,000 per patient [17,18].

The research examined the direct and indirect costs associated with sickle cell disease (SCD) in Saudi Arabia, encompassing 217 participants aged 18 and older. The findings indicated significant societal expenses, with an average cost per patient of USD 48,506. The average cost of healthcare, which includes hospital stays, informal care, and medication, was USD 21,415 [19].

According to the September 2021 report by the general evaluation of Pillars of Spending Sustainability Program implanted by the Expenditure and Projects Efficiency Authority (EXPRO), the Ministry of Health was recognized for its top ranking among ministries of the Kingdom of Saudi Arabia [20]. This recognition emphasizes the health system’s commitment to such programs, aligning with goals of the Kingdom’s Vision 2030. The primary aim is to establish a balanced and efficient healthcare system that caters to both medical and economic aspects, ensuring quality health standards while minimizing financial inefficiencies.

Efforts to reduce financial waste serve the purpose of enabling the provision of advanced healthcare. This, in turn, contributes to the success of the healthcare system in delivering optimal care to all participants. Maximizing the effectiveness of counseling and disease prevention clinics plays a key role in achieving the objectives of promoting healthy marriages, EXPRO, and eventually 2030 Vision goals.

This study aims to estimate the costs related to the government’s mandatory premarital screening for sickle cell disease and compare these costs with those resulting from high-risk marriages, as well as government healthcare spending on sickle cell disease management. This study also evaluates the health and economic outcomes of the PMS program in the Saudi health system.

## 2. Materials and Methods

This evaluation was focused on tangible costs within the healthcare system. The approach was conducted from a healthcare perspective, emphasizing data related to specific expenses, including PMS costs, and disease management costs.

### 2.1. Model Development

**Figure 1:**
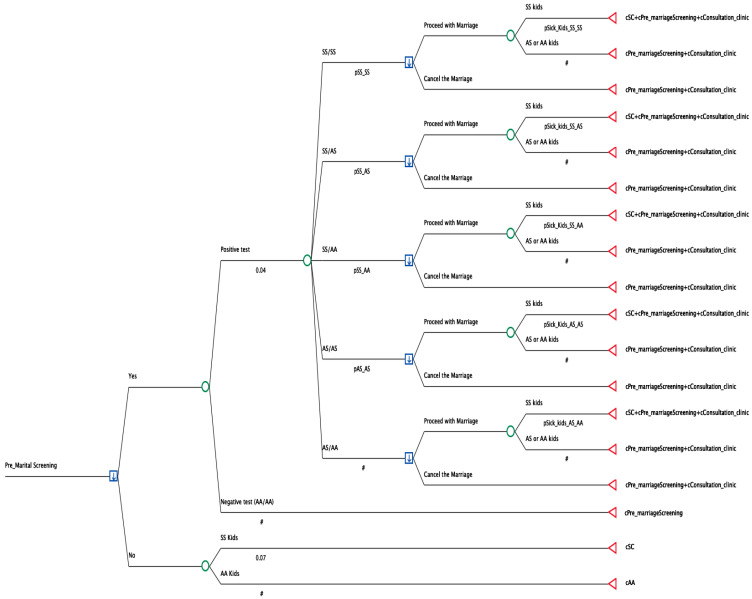
Decision tree model.

A decision tree model was conceptualized to delineate the potential pathways stemming from premarital screening (PMS) procedure. Figure 1 illustrates the structure of this decision tree. To facilitate comparison, 300,000 cases in each branch were analyzed.

At the starting point, intending couples undergo screening, which typically results in either a positive or negative outcome. A negative result requires no further action. However, in the case of positive tests, the subsequent stage involves identifying the specific type of positive test (couple incompatibility), which may include the following possible scenarios:

− Sickle cell disease and Sickle cell disease (SS/SS);
− Sickle cell disease and Sickle cell trait (SS/AS);
− Sickle cell disease and Healthy hemoglobin (SS/AA);
− Sickle cell trait and Healthy hemoglobin (AS/AA);
− Sickle cell trait and Sickle cell trait (AS/AS).

Each of these five possibility branches is further tracked into potential marriage, having children, and the health status of their children. The probabilities and percentages were calculated based on epidemiological data from a previously published PMS report [7,8,21]. These data provided insights into the modeled costs associated with disease management for children with sickle cell disease and those with sickle cell traits. The primary outcomes measured were the model costs obtained from premarital screening and the yearly model costs of managing sickle cells in the population. The PMS data were obtained from the previously published literature, and the costs of managing sickle cells had a one-year perspective [19,22,23]. A direct cost comparison is used to show cost savings or otherwise.

### 2.2. Variables/Input

Transition probabilities were calculated based on data obtained from previously published studies and are detailed in Table 1. The yearly treatment costs for sickle cell disease were estimated using international cost data. There were no costs computed for treating those with sickle cell traits (the carriers) because they relatively live healthy lives and are rarely sick due to sickle cell disease. Essential variables pertaining to sickle cell disease were compiled and organized in Excel spreadsheets using Microsoft Office Excel (2019). These data included probabilities associated with sickle cell disease (both trait and disease), probabilities of high-risk marriages, and probabilities of high-risk couples who decided to proceed with marriage. The dataset also included the estimated costs of the premarital screening test and subsequent consultation fees in the event of positive test results. To ensure accuracy, the highest possible prices for premarital examinations and counseling clinics at major city hospitals were used in comparison with the annual treatment costs for sickle cell disease patients. The annual management cost for sickle cell disease was approximated based on the medical literature and an experts’ opinion.

A decision analytic model was constructed using TreeAge Pro HEALTHCARE 2022 Software to visually represent the collected information. Considering the duration of the study is one-year horizon, discounting was not applied. The inputs of various variables are delineated in Table 1.

### 2.3. Sensitivity Analysis

To explore parameter uncertainty, one-way sensitivity analyses were conducted on all key model inputs. Specifically, we varied the costs of screening testing, consultation fees, and the cost for sickle cell disease (SCD) management. For variation ranges, we applied a variance of ±25% around the base-case estimate. It enabled us to determine the parameters that impact the overall outcome and the robustness of the base-case findings. The results are presented using a tornado diagram, which highlights the parameters that have the greatest impact on model outcomes.

## 3. Results

### 3.1. Base Results

The decision tree model considered the costs of premarital screening vs. not screening. The expected value (EV) of cost per pair for the screening method was USD 308, but for the no-screening strategy it was USD 1928. This suggests that the screening approach saved a significant amount of money.

Within the screening branch, couples that tested positive were classified into five genetic groups: SS/SS, SS/AS, AS/AS, SS/AA, and AS/AA. The estimated expenses for these subdivisions were USD 27,000, USD 13,813, USD 7156, USD 500, and USD 500, respectively (Figure S1, Supplementary Materials). These data indicate that high-risk combinations (SS/SS and SS/AS) are the most expensive, whereas low-risk genotypes are the cheapest.

Negative tests incur no further costs, as they pose no risk of having sickle cell disease or traits. These negative tests had an estimated cost of USD 300. From the healthcare perspective, the annual savings from preventing high-risk couples from proceeding to marriage are approximately USD 10,746,450 (Table 2)

A direct comparison to the no-screening arm when running 300,000 couples through each arm reveals that the modeled PMS arm cost is USD 3,696,000 where no screening is USD 40,488,000, which represents roughly a 73% reduction in the overall healthcare costs if no screening is implemented (Table 3).

### 3.2. Sensitivity Analysis Results

Tornado diagrams demonstrated that key variables influencing the incremental expected values (EVs) for decision nodes related to outcomes involving children with sickle cell disease are the costs associated with treating children having sickle cell disease, the cost linked to premarital screening tests, and the consultation clinic costs for partners testing positive.

A one-way sensitivity analysis showed that varying the total cost of sickle cell management resulted in incremental expected values ranging from USD 1153.7 to USD 2085.6. A summary of these results is presented in Figures S2–S4 in the Supplementary Materials.

## 4. Discussion

This economic impact study assesses the costs and potential risks that high-risk marriages, particularly in the context of sickle cell disease, might pose on healthcare spending and outcomes. A direct comparison between healthcare costs associated with managing sickle cell disease and the costs incurred in a premarital screening program demonstrates direct cost savings. Premarital screening not only offers cost savings but also provides significant health benefits by allowing couples to understand their sickle cell status. It models the potential health impact if intending couples decide to proceed with marriage. From a healthcare cost perspective, the most favorable scenarios involve marriages between a trait carrier and a healthy partner and marriages between a sickle cell partner and a healthy one. In these scenarios, the children are unlikely to have SCD; at most, the trait is inherited in a recessive form, which substantially limits the long-term clinical and economic burden.

The cost modeled for managing sickle cell conditions through a premarital screening involving 300,000 couples is USD 10,746,450 compared with USD 40,488,000 for a year without screening, implying an absolute difference of approximately USD 29.74 million and a 73% reduction in healthcare spending with PMS.

Additionally, the model’s results indicate a significant reduction in EVs in the PMS arm despite the prevalence of high-risk couples proceeding with marriages. This demonstrates that the premarital screening program is a cost-saving and efficient method. One-way sensitivity analyses support the robustness of these findings: as total SCD management costs vary, incremental EVs remain within USD 1153.7–USD 2085.6, preserving both the direction and magnitude of the savings. These estimates are consistent with the high direct medical and system-level costs of SCD reported in the Saudi context and the broader region, underscoring the sizeable economic headroom for prevention-focused strategies [19,22].

The study also reflects real-world behavior, as over 90% of couples with sickle cell disease or related traits proceed with marriage, consistent with Alswaidi et al. (2009), who found that 89.6% of high-risk couples proceeded with their marriage plans [3]. Factors influencing this decision include apprehension about starting a new relationship, difficulty in canceling wedding arrangements, societal acceptability, and family influences [8,21]. Additionally, recent systematic reviews and epidemiological studies confirm that sickle cell disease (SCD) remains relatively common in Saudi Arabia, with prevalence rates ranging from 2% to 2.6% in some regions and significantly higher carrier rates, particularly in the Eastern and Southern provinces [5,7,25]. To maximize the impact of PMS, policymakers should prioritize expanding and tailoring screening programs in regions with high prevalence of the disease. This targeted approach will ensure that resources are effectively allocated, thereby enhancing the overall benefits of the screening program. However, there is a strong consensus that the prevalence of SCD is anticipated to decrease over the next decades, primarily due to the implementation and expansion of premarital screening programs and increased public awareness [25].

The study findings have significant implications for public health policy in Saudi Arabia. Identifying high-risk couples and preventing affected births through PMS can reduce the healthcare burden and support Saudi Vision 2030’s goals of improving population health, reducing preventable diseases, and enhancing system efficiency [12]. By demonstrating the cost-effectiveness of PMS and identifying key factors that influence outcomes, our study provides actionable evidence to inform national policy and resource allocation for genetic screening initiatives. The healthcare system should investigate why individuals proceed with high-risk marriages despite screening results. Expanding screening to adults of potential partnership age can increase awareness of sickle cell status before a relationship is formed. Additionally, public education efforts should be strengthened to communicate the risks of entering marriage when a high genetic risk is identified.

Several limitations are present in this analysis. The model excludes the costs associated with managing adults diagnosed with sickle cell disease (SCD) and does not consider occasional medical events among carriers. It assumes high continuation rates in high-risk marriages, which may not accurately represent real-world behavior and could lead to an overestimation of the number of affected births prevented. The analysis employs a one-year cost horizon for downstream SCD management, focusing solely on direct healthcare costs and omitting indirect or societal costs, such as productivity losses, caregiver time, and broader social impacts. Including these factors could influence the economic evaluation. Moreover, the study’s results are adjusted to the Saudi context by utilizing local costs, cultural assumption, and national PMS data. Since the results are in support of the PMS policy in Saudi Arabia, generalization to other countries should take into consideration variations in the prevalence of genetic disorders, the cost of treatment, and public perceptions of premarital counseling.

The model was developed specifically for cost estimation and direct comparison of sickle cell costs. Its strengths include a transparent decision tree structure that covers all relevant parental genotype pairings, clearly defined transition probabilities, and systematic one-way sensitivity analyses on high-influence parameters such as SCD cost, screening cost, and counseling cost, which support the robustness of the cost-saving conclusion.

## 5. Conclusions

In Saudi Arabia, the premarital screening program is an important component of public health. It significantly reduces healthcare costs by 73%, prevents severe genetic disorders like SCD, and supports the nation’s long-term health vision. It appears that PMS is an efficient strategy that decision makers cannot afford to overlook. The findings of this study show the significant clinical and economic benefits of PMS. These benefits include immediate financial savings as well as a reduction in the long-term burden of genetic disorders imposed on families and healthcare systems; hence, PMS allows high-risk couples to make educated reproductive decisions, improving the quality of life for future generations. Expanding and strengthening PMS will be essential for building a resilient and healthy society as Saudi Arabia progresses toward its Vision 2030 goals. Policymakers should raise public awareness and invest in research to ensure PMS remains effective and responsive to changing demographic and healthcare needs. These actions will contribute to long-term health security and sustainability while reinforcing Saudi Arabia’s leadership in public health.

## Figures and Tables

**Table 1:** List of Variables/Inputs.

NAME	DESCRIPTION	Value [Reference]
cConsultation_clinic	Cost of consultation clinic for partner with positive test	USD 200 *
cPre_marriageScreening	Cost of pre-marriage screening test	USD 300 *
cSC	Cost of treating sickle cell case	USD 26,626 [19,22]
cAA	Average healthcare expenditure per capita	USD 68.60 [23]
pPostitveTest	Probability of having positive premarital test (Diseased or trait)	0.045 [7,21]
pSS_AA	Probability of SS/AA couple	0.025 [8]
pSS_SS	Probability of SS/SS couple	0.025 [8]
pSS_AS	Probability of SS/AS couple	0.025 [8]
pSick_Kids_SS_AA	Probability of having sickle cell kid from SS/AA parents	0 [24]
pSick_Kids_SS_SS	Probability of having sickle cell kid from SS/SS parents	1 [24]
pSick_kids_SS_AS	Probability of having sickle cell kid from SS/AS parents	0.5 [24]
pSick_kids_AS_AA	Probability of having sickle cell kid from AS/AA parents	0 [24]
pSick_kids_AS_AS	Probability of having sickle cell kid from AS/AS parents	0.25 [24]
pNegative test	Probability of having a negative test	0.953 [7,21]
pProceeding_with_marraige	Probability for couple with positive test to proceed with marriage	0.77 [8,21]

* Estimated based on highest local private hospital prices and expert opinion.

**Table 2:** Yearly healthcare costs saving if high-risk marriages were canceled.

Healthcare Perspective	Value	Reference
Sample population	300,000	[9]
Costs of managing sickle cell disease	USD 26,626	[22]
SS/SS genotype marriage canceled	USD 8,137,800	calculated
SS/AS genotype marriage canceled	USD 2,071,950	calculated
AS/AS genotype marriage canceled	USD 536,700	calculated
**Total costs saving**	**USD 10,746,450**	

**Table 3:** Yearly healthcare costs modeled for each strategy.

Strategy	EV *	Cost
Screening	USD 308	USD 10,746,450
No screening	USD 1928	USD 40,488,000
Incremental difference	USD 1620	USD 29,741,550

* EV: expected value.

## Data Availability

The original contributions presented in this study are included in the article/Supplementary Materials. Further inquiries can be directed to the corresponding authors.

## Supplementary Materials

The following supporting information can be downloaded at: https://www.mdpi.com/article/10.3390/healthcare13172243/s1.

## References

[1] Ministry of Health Premarital Screening. https://www.moh.gov.sa/en/HealthAwareness/Beforemarriage/Pages/default.aspx.

[2] Petrou M. (2013). Genetic Counselling. Prevention of Thalassaemias and Other Haemoglobin Disorders: Volume 1: Principles [Internet].

[3] Alswaidi F.M., O’brien S.J. (2009). Premarital Screening Programmes for Haemoglobinopathies, HIV and Hepatitis Viruses: Review and Factors Affecting Their Success. J. Med. Screen.

[4] Executive Board, 118 Thalassaemia and Other Haemoglobinopathies: Report by the Secretariat, 2006, Executive Board 118th Session, Provisional Agenda Item 5.2. https://iris.who.int/handle/10665/21519.

[5] Jastaniah W. (2011). Epidemiology of Sickle Cell Disease in Saudi Arabia. Ann. Saudi Med..

[6] Memish Z.A., Owaidah T.M., Saeedi M.Y. (2011). Marked Regional Variations in the Prevalence of Sickle Cell Disease and β-Thalassemia in Saudi Arabia: Findings from the Premarital Screening and Genetic Counseling Program. J. Epidemiol. Glob. Health.

[7] Aljabry M., Sulimani S., Alotaibi G., Aljabri H., Alomary S., Aljabri O., Sallam M., Alsultan A. (2024). Prevalence and Regional sDistribution of Beta-Hemoglobin Variants in Saudi Arabia: Insights from the National Premarital Screening Program. J. Epidemiol. Glob. Health.

[8] Memish Z.A., Saeedi M.Y. (2011). Six-Year Outcome of the National Premarital Screening and Genetic Counseling Program for Sickle Cell Disease and β-Thalassemia in Saudi Arabia. Ann. Saudi Med..

[9] Alsaeed E.S., Farhat G.N., Assiri A.M., Memish Z., Ahmed E.M., Saeedi M.Y., Al-Dossary M.F., Bashawri H. (2018). Distribution of Hemoglobinopathy Disorders in Saudi Arabia Based on Data from the Premarital Screening and Genetic Counseling Program, 2011–2015. J. Epidemiol. Glob. Health.

[10] Umair M., Alfadhel M. (2020). Prevention of Hemoglobinopathies in Saudi Arabia: Efficacy of National Premarital Screening and the Feasibility of Preimplantation Genetic Diagnosis. J. Biochem. Clin. Genet..

[11] Alotaibi M.M. (2017). Sickle Cell Disease in Saudi Arabia: A Challenge or Not. J. Epidemiol. Glob. Health.

[12] Gosadi I.M. (2019). National Screening Programs in Saudi Arabia: Overview, Outcomes, and Effectiveness. J. Infect. Public Health.

[13] Premarital Screening—Accredited Premarital Screening Centers. https://www.moh.gov.sa/en/HealthAwareness/Beforemarriage/Pages/002.aspx.

[14] Abu-Shaheen A., Heena H., Nofal A., Abdelmoety D.A., Almatary A., Alsheef M., AlFayyad I. (2020). Epidemiology of Thalassemia in Gulf Cooperation Council Countries: A Systematic Review. BioMed Res. Int..

[15] Huo J., Xiao H., Garg M., Shah C., Wilkie D.J., Iii A.M. (2018). The Economic Burden of Sickle Cell Disease in the United States. Value Health.

[16] Baldwin Z., Jiao B., Basu A., Roth J., Bender M.A., Elsisi Z., Johnson K.M., Cousin E., Ramsey S.D., Devine B. (2022). Medical and Non-Medical Costs of Sickle Cell Disease and Treatments from a US Perspective: A Systematic Review and Landscape Analysis. PharmacoEconomics Open.

[17] Shah N., Bhor M., Xie L., Paulose J., Yuce H. (2020). Medical Resource Use and Costs of Treating Sickle Cell-Related Vaso-Occlusive Crisis Episodes: A Retrospective Claims Study. J. Health Econ. Outcomes Res..

[18] Holdford D., Vendetti N., Sop D.M., Johnson S., Smith W.R. (2021). Indirect Economic Burden of Sickle Cell Disease. Value Health.

[19] Shdaifat E., Abu-Sneineh F., Alsaleh N., Ibrahim A. (2025). Economic Burden of Sickle Cell Disease in Saudi Arabia. Value Health Reg. Issues.

[20] Ministry of Health MOH News. MOH Obtains First Place in Institutional Excellence in Spending Efficiency. https://www.moh.gov.sa/en/Pages/Default.aspx.

[21] AlHamdan N.A., AlMazrou Y.Y., AlSwaidi F.M., Choudhry A.J. (2007). Premarital Screening for Thalassemia and Sickle Cell Disease in Saudi Arabia. Genet. Med..

[22] AlRuthia Y. (2025). The Direct Medical Costs of Sickle Cell Disease in Saudi Arabia: Insights from a Single Center Study. Healthcare.

[23] The United Nations Development Programme (UNDP) (2019). The Cost of Health Services Delivered at Primary Care Facilities in SAUDI ARABIA. www.undp.org/sites/g/files/zskgke326/files/2024-02/ksa_phc_costing_report.pdf.

[24] Sheehan V.A., Gordeuk V.R., Kutlar A., Kaushansky K., Prchal J.T., Burns L.J., Lichtman M.A., Levi M., Linch D.C. (2021). Disorders of Hemoglobin Structure: Sickle Cell Anemia and Related Abnormalities. Williams Hematology, 10e.

[25] Asraf N.O., Aljibreen S.A., Alotaibi M.B., Hadadi M.H., Ahmed H.A.F., Batwie A.A., Alghamdi W.M., Al-Jaffer Y.H., Alhai S.M., Alshehab K.A. (2022). Prevalence of Sickle-Cell Disease in Saudi Arabia: A Systematic Review. Uttar Pradesh J. Zool..

