# Supplementary material for: The Economic Impact of Premarital Screening (PMS) of Sickle Cell Anemia on the Saudi Health System: A Cost Analysis Study
Source: Healthcare (Basel). 2025 Sep 8;13(17):2243. doi: 10.3390/healthcare13172243 (PMC12428215; doi:10.3390/healthcare13172243)

Figure S-1: Decision tree with Expected values

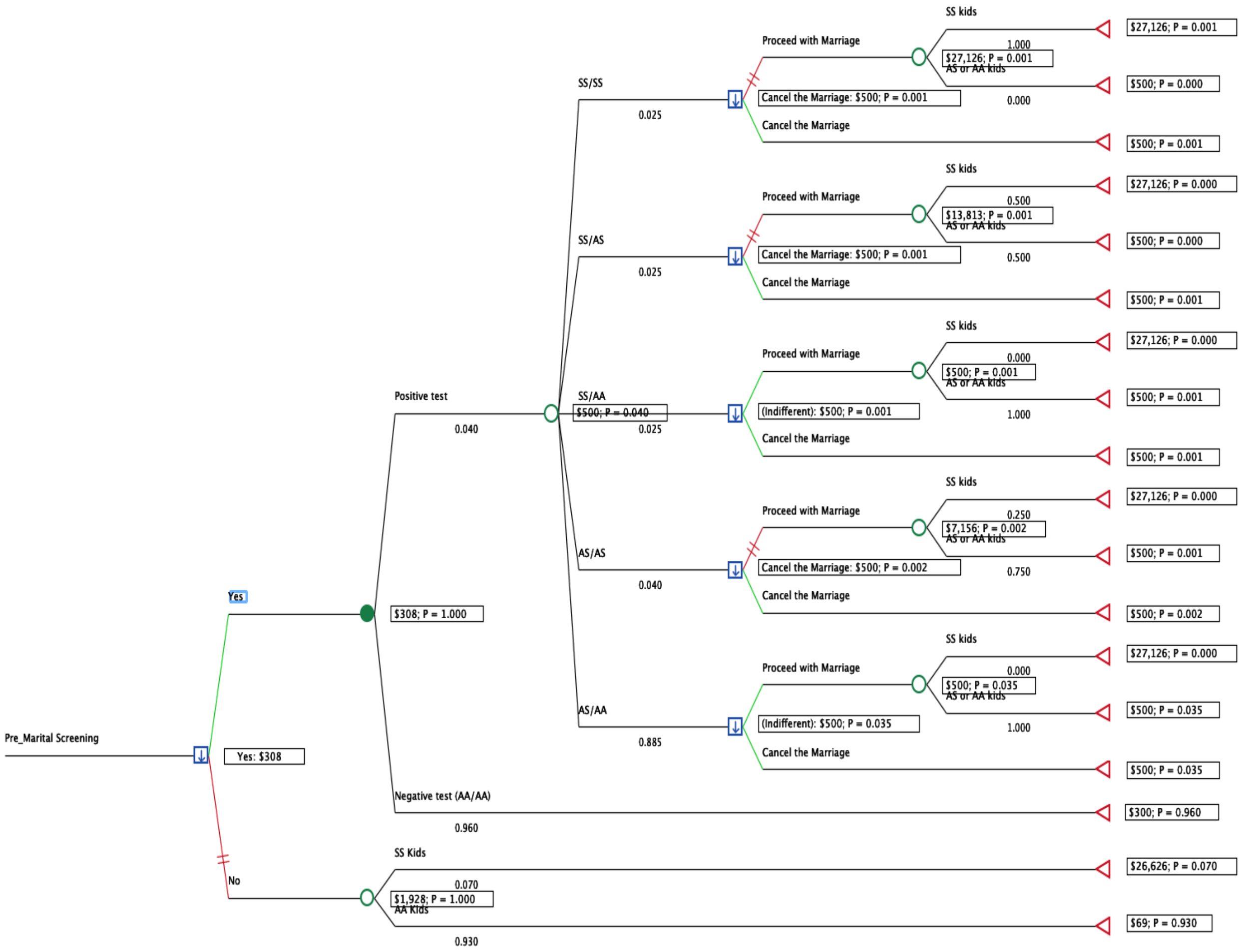

Figure S-2: Tornado Diagram- Incremental EVs

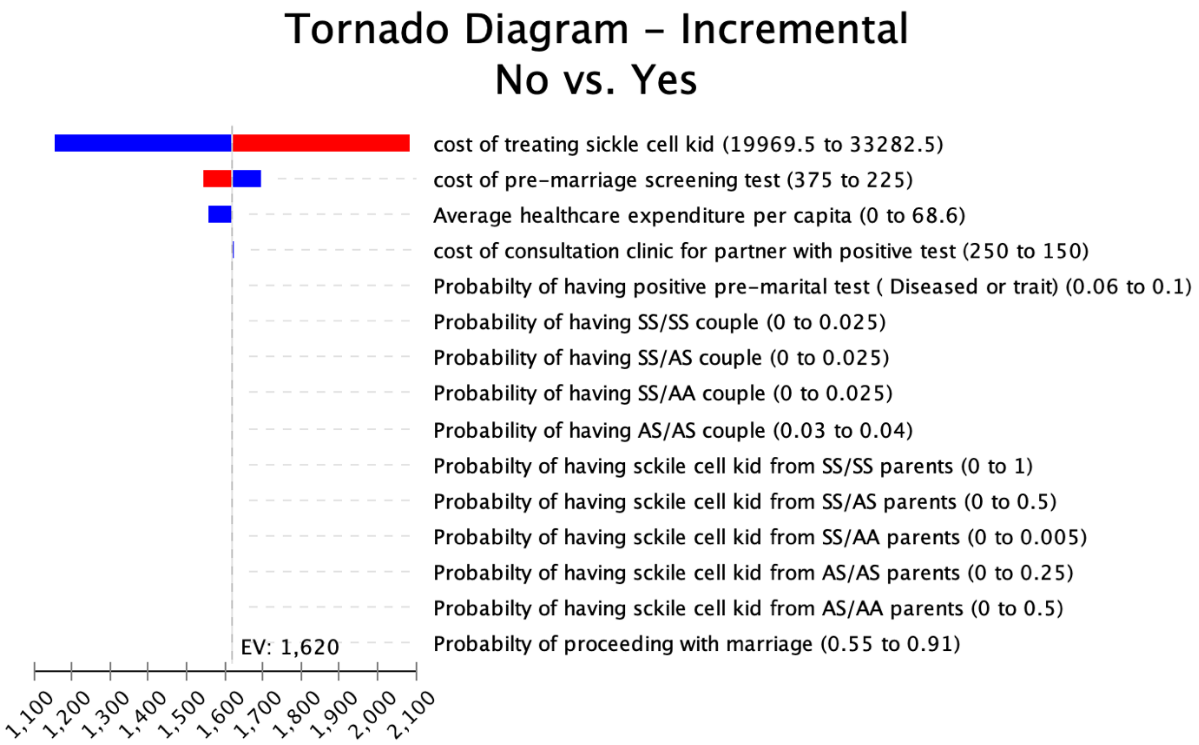

Figure S-3: 1-Way Sensitivity Analysis for treatment cost for Sickled cell

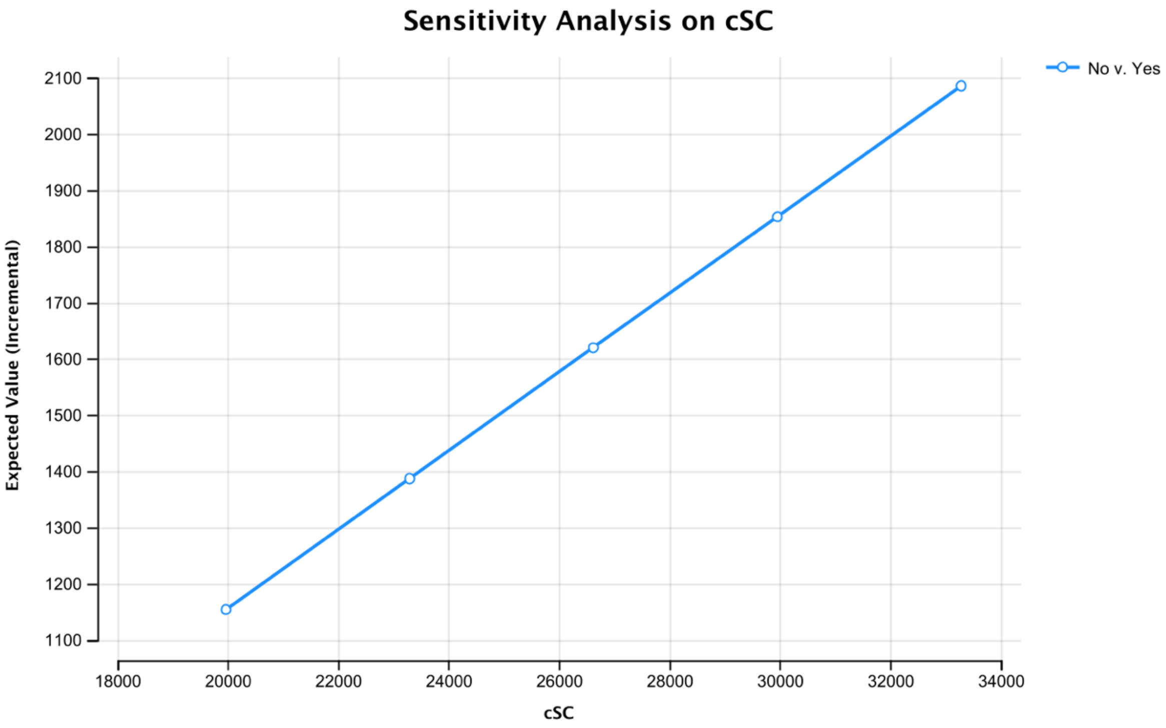

Figure S-4: 1-Way Sensitivity Analysis for treatment cost consultation clinic

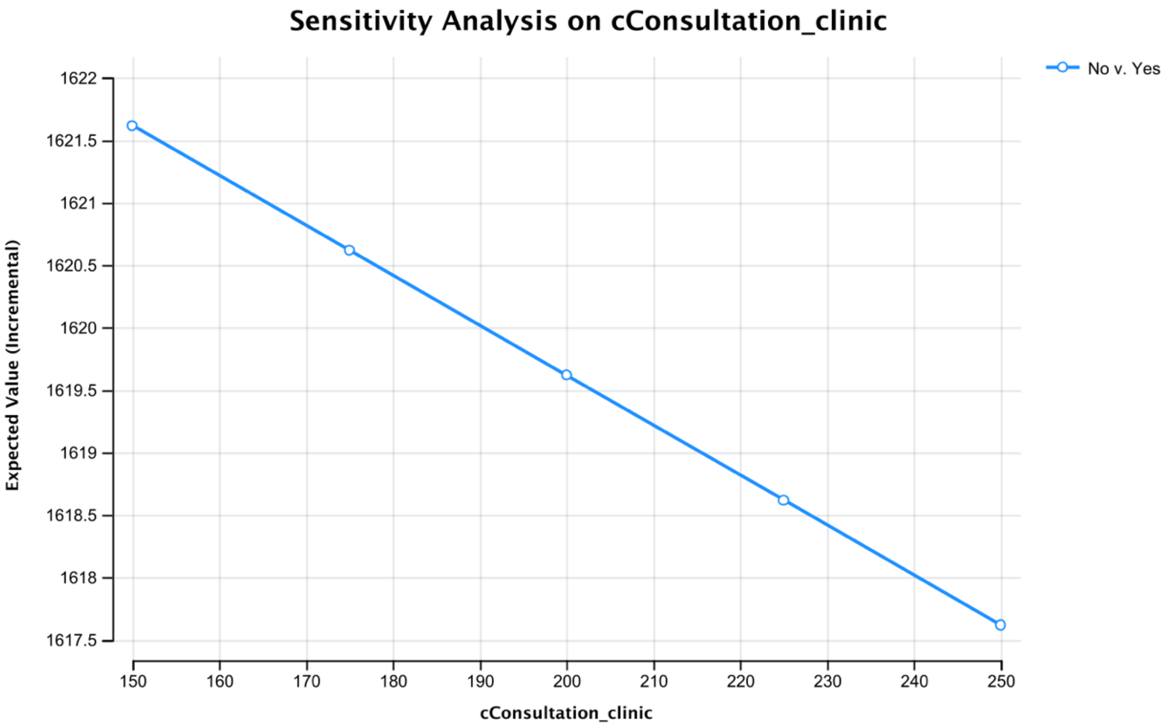

Supplement: Supplementary file 1 [file healthcare-13-02243-s001.zip › healthcare-3786064-supplementary.pdf]
